# Health promotion, the social determinants of health, and urban health: what does a critical discourse analysis of World Health Organization texts reveal about health equity?

**DOI:** 10.1186/s44263-023-00023-4

**Published:** 2023-12-01

**Authors:** Michelle Amri, Theresa Enright, Patricia O’Campo, Erica Di Ruggiero, Arjumand Siddiqi, Jesse Boardman Bump

**Affiliations:** 1 School of Public Policy, Simon Fraser University, Harbour Center, 515 West Hastings Street, Vancouver, BC V6B 4N6 Canada; 2 Takemi Program in International Health, Harvard T.H. Chan School of Public Health, Harvard University, 665 Huntington Avenue, Bldg. 1, Boston, MA 02115-6021 USA; 3 Social and Behavioural Health Sciences Division, Dalla Lana School of Public Health, University of Toronto, 155 College Street, Toronto, ON M5T 1P8 Canada; 4 Department of Political Science, University of Toronto, 100 St George Street, Toronto, ON M5S 3G3 Canada; 5 Division of Epidemiology, Dalla Lana School of Public Health, University of Toronto, 155 College Street, Toronto, ON M5T 1P8 Canada; 6 Li Ka Shing Knowledge Institute, St. Michael’s Hospital, 209 Victoria Street, Toronto, ON M5B 1T8 Canada; 7 Gillings School of Global Public Health, University of North Carolina-Chapel Hill, Chapel Hill, USA; 8 Department of Social and Behavioral Sciences, Harvard T. H. Chan School of Public Health, Harvard University, Boston, USA; 9 Bergen Centre for Ethics and Priority Setting, University of Bergen, Bergen, Norway

**Keywords:** Health equity, Equity, Health inequity, Inequity, Global health, Public health, Health policy, Public policy, World Health Organization, Critical discourse analysis

## Abstract

**Background:**

The World Health Organization (WHO) has focused on health equity as part of its mandate and broader agenda—consider for example, the “health for all” slogan. However, a recent scoping review determined that there are no studies that investigate the WHO’s approach to health equity. Therefore, this study is the first such empirical analysis examining discourses of health equity in WHO texts concerning health promotion, the social determinants of health, and urban health.

**Methods:**

We undertook a critical discourse analysis of select texts that concern health promotion, the social determinants of health, and urban health.

**Results:**

The findings of this study suggest that (i) underpinning values are consistent in WHO texts’ approach to health equity; (ii) WHO texts reiterate that health inequities are socially constructed and mitigatable but leave the ‘causes of causes’ vague; (iii) despite distinguishing between health “inequities” and “inequalities,” there are several instances where these terms are used interchangeably across texts; (iv) WHO texts approach health equity broadly (covering a variety of areas); (v) health equity may be viewed as applicable either throughout the life-course or intergenerationally, which depends on the specific WHO text at hand; and (vi) WHO texts at times use vague or unclear language around how to improve health equity.

**Conclusions:**

This study does not present one definition of health equity and action to be taken. Instead, this study uncovers discourses embedded in WHO texts to spur discussion and deliberate decision-making. This work can also pave the way for further inquiry on other complex key terms or those with embedded values.

**Supplementary Information:**

The online version contains supplementary material available at 10.1186/s44263-023-00023-4.

## Background

The World Health Organization (WHO) has long emphasized health equity as a central tenet of its work. In the Declaration of Alma-Ata in 1978 [1], for example, the WHO explicitly underscores its goal of “promot[ing] the health of all the people of the world.” More recently, this focus on health equity has been emphasized in the work of the WHO’s Commission on the Social Determinants of Health (CSDH; [2]). However, what health inequity has meant in practice is not entirely clear. In response, the WHO commissioned Margaret Whitehead to define inequity in health in 1990. According to Whitehead, inequity in health equated to “differences which are *unnecessary* and *avoidable* … [and] considered *unfair* and *unjust*” [3]; a definition that has been praised as accessible, concise, intuitive, and easily communicated [4]. Although this definition has been widely accepted and used internationally, scholars have noted there is ambiguity respecting the distinctions between health inequity, health inequalities, and health disparities [4, 5]. This ambiguity is apparent both in the definition of health inequity and its operationalization, a dilemma that is not specific to the WHO. Although *health inequity* entails a normative assumption that inequities are unfair, *health inequality* is a measured difference [6]: that is, a descriptive definition that does not entail being unfair. However, it is immensely difficult to determine what is in fact a health inequity as opposed to an inequality. Characterizations of health inequity (e.g., unnecessary, unfair) are open to varying interpretations which can be problematic [4, 7]. To illustrate, differences can arise if one policymaker understands *unfair* to mean with respect to counterparts in a similar socioeconomic position (SEP) or socioeconomic status (SES) [8, 9] in the same city, whereas another may interpret unfair to mean with respect to the population-at-large; or if one policymaker understands *avoidable* as meaning a health inequity can be remedied through the healthcare system, whereas another understands this to mean a change in policies affecting the social determinants of health, and yet another through changing the political climate itself. In the context of health, then, the definition of equity has consequences for its operationalization [10], as these differing understandings result in different approaches to policy and practice. This is problematic for both how health equity is understood and for the execution of subsequent action (e.g., implications for measurement and accountability [5]).

In response to identified ambiguities, a recent scoping review of the WHO’s approach to equity [11] was undertaken to systematically search the peer-reviewed literature to understand how equity has been referred to and its conceptual underpinning [12]. This review determined that the WHO has held—and continues to hold—ambiguous, inadequate, and contradictory views of equity [12]. For example, some scholars felt that the WHO approaches health equity through largely focusing on SES, whereas other times the WHO focuses on various facets of inequity. It is noteworthy that this scoping review found no empirical articles, of either a quantitative or qualitative nature, assessing the WHO’s interpretations and approaches to equity, despite not restricting the search. Given these ambiguities, our study seeks to fill this gap in the research and empirically examine how the WHO conceptualizes health equity by conducting a critical discourse analysis (CDA) of select texts concerning health promotion, the social determinants of health (SDH), and urban health. CDA, a method and methodology used in the social sciences, is employed because it can help tease out how health equity is used in WHO texts by assessing what language is used, how it is used, and what is not being stated. Through this empirical analysis, we aim not only to arrive at a more nuanced understanding of health equity, but also to unveil understandings of implicit normative positions that are reflective of the WHO’s work. And not only the WHO’s work, but other global health work, given the overarching power the WHO possesses and its role to act as the “directing and co-ordinating authority on international health work”, as outlined in its constitution [13].

## Methods

### Critical discourse analysis

CDA is a method and methodology that investigates how phenomena are discussed. Given that values are inherent in the WHO’s approach to health equity, CDA is one method that rejects value-free science [14]. CDA allows for building on multiple understandings and interpretations of health equity by centering inquiry around discourses, which can be defined broadly as “anything beyond the sentence, language use, and a broader range of social practice that includes non-linguistic and non-specific instances of language” [15]. The WHO’s discourses are worthy of study given that the WHO is regarded as *the* authoritative voice on global health, and how it writes and talks about phenomena has a significant impact on individual attitudes and behaviors as well as public policies.

Because CDA can allow for the understanding of discourses [14], uncovering underlying narratives of ideologies and claims, and the identification of contradictions, gaps, and unrealized possibilities for change [16], it affords a well-suited method and methodology for this study. The WHO, as an organization, is made up of multiple intersecting parts and processes—and these dynamics ultimately shape how the WHO can and does speak about phenomena. Additionally, CDA was selected for this study because it affords a critical perspective that ultimately seeks to combat inequity [14]. Overall, this study employs a non-prescriptive methodology to read texts closely to uncover discourses and present insights about health equity that other methods do not afford [17].

### Approach to analysis

CDA neither has a unitary theoretical framework nor denotes a specific approach to conducting research [14] or a specific sampling procedure [18]. With this approach of assessing language use in texts, the texts themselves constitute data in the analysis (Table 1). Each datum was analyzed by MA using both a priori, or deductive, and inductive codes. A priori codes—“equity or inequity” and “equality or inequality”—facilitated the analysis of how these terms are used. In addition, applying the question “equality of what?”, famously posed by Sen [19], or a variation of “health inequities in what?” allowed for further insights when investigating the a priori codes. Inductive codes—such as “intergenerational,” “what is inequity?”, and “indirect or unclear”—emerged from careful reading of texts to observe aspects not covered by the a priori codes, which allowed for enhanced consideration of discourses. Both a priori and inductive codes facilitated the categorizing of language and the reassessment of these categories through additional data when new discourses presented—in line with CDA [18]. Analysis was conducted across texts and not as a distinct list per datum, which guided analysis and the construction of the findings section. This exploratory discursive analysis was not conducted with the intention of weighing or ranking texts and their relationship to these discourses, or to uncover findings along set themes in a framework. Instead, the goal was to analyze discourses. “Positionality”, drawn from critical social science research, implies that one’s position may influence various aspects of the research, such as in collecting or interpreting data [20]. Thus, the primary coder (MA) and her positionality is understood to influence analysis. She is a social science researcher in global and public health with experience consulting for the WHO. She led the scoping review focused on the WHO’s approach to equity [11], and thus, her experience has informed this CDA. The positionality of all authors whose works align with the critical tradition as opposed being squarely situated in biomedical science also influence this study. As MA presented emergent discourses with illustrative quotes from the texts analyzed, the entire authorship team reviewed the coded text and corresponding findings and discussed discrepancies to reach consensus. NVivo 12 software was used to code the texts.

**Table 1:** List of WHO text data sources

	Source	PDF pages
1	*Closing the gap in a generation* [2]	256
2	*Our cities, our health, our future* [21]	199
3	*Equity, social determinants and public health programmes* [22]	303
4	*Urban HEART: Urban Health Equity Assessment and Response Tool* [23]	48
5	*Urban HEART User Manual* [24]	59
6	*Hidden Cities: Unmasking and Overcoming Health Inequities in Urban Settings* [25]	145
7	*Rio Political Declaration on Social Determinants of Health* [26]	7
8	*Global report on urban health: equitable, healthier cities for sustainable development* [27]	241
9	*Promoting health in the SDGs, Report on the 9th Global Conference for Health Promotion: All for health, health for all* [28]	44
Total		1302

### Data sources

**Fig. 1:**
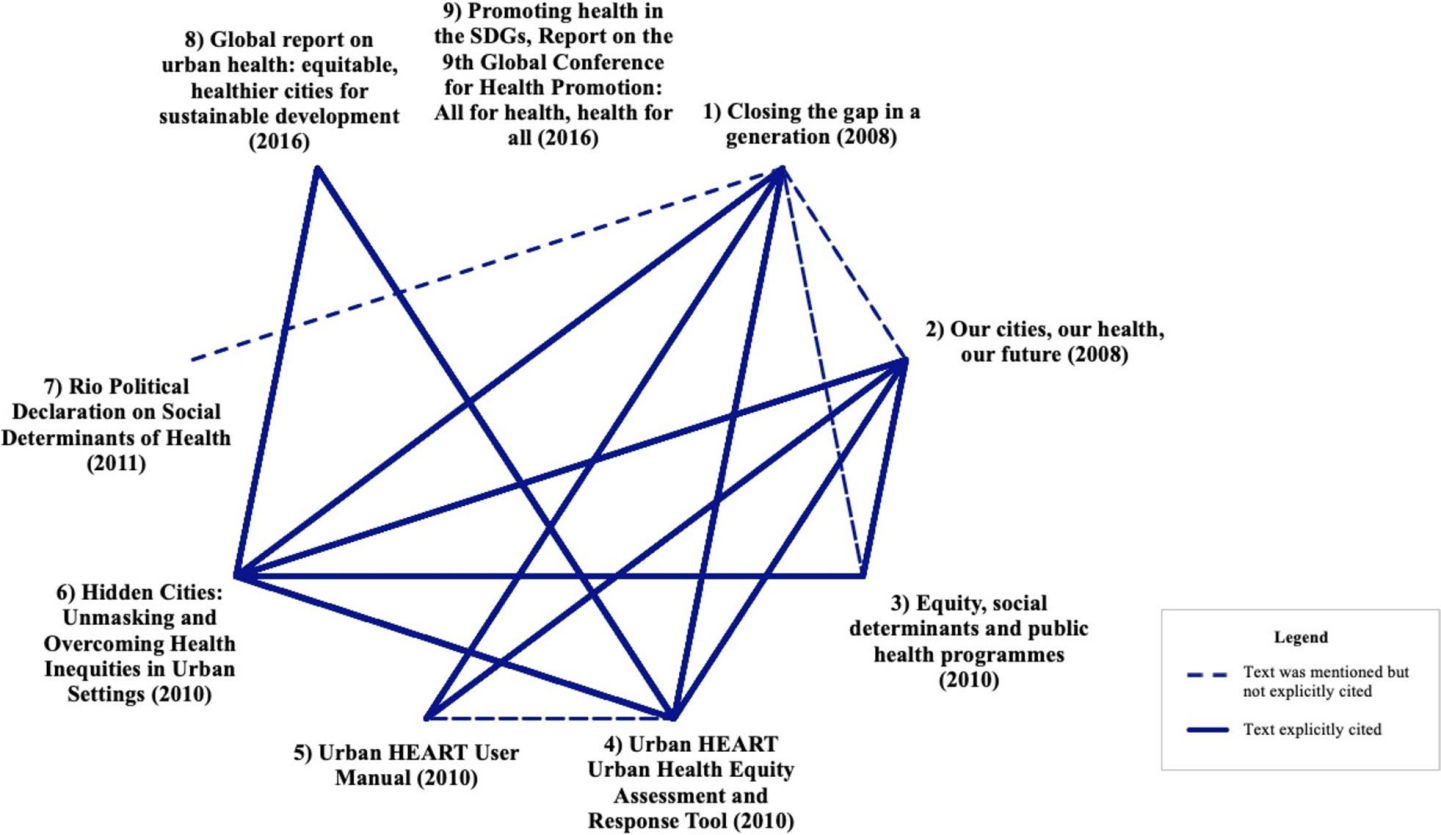
Depiction of interrelations: cross-referencing between selected texts

The data sources, listed in Table 1, concern health promotion, the SDH, and urban health. These three domains were selected because they are cross-disciplinary, focus on upstream solutions, and provide different perspectives on health equity. These three areas are relatively easy to compare because they are all related. Health promotion attempts to address broad determinants of health inequity. The SDH reflect an upstream perspective. And urban health was selected because cities are where inequities are greatest. Within these domains, we used a multi-step process to select texts. We began with search results from a scoping review on the WHO’s concept of equity in health that yielded 2558 hits [12]. We reviewed these results keeping a list of WHO texts that were cited, discussed, or mentioned. In tracking these results, we focused our attention on texts appearing multiple times and focused on health equity, an approach which allowed us to narrow our analysis. We applied several criteria for our assessment. We included texts published from 2008 until 2021, when the CDA was conducted. Given the influence of the CSDH’s work, particularly in terms of shifting understanding around the SDH [29], the CSDH’s final report was the first text included in this study, with all the preceding reports being excluded. Texts from CSDH Knowledge Networks, such as the Early Child Development or Employment Conditions Knowledge Networks, were excluded because their direct focus is outside of the three outlined domains. The only exceptions were the texts from the Knowledge Network on Urban Settings, which focused on urban health, and the Priority Public Health Conditions Knowledge Network, which focused on equity and the SDH explicitly in the title of the included text. We assessed the influence of WHO texts based on the number of times texts were mentioned in the search results. For example, the *Rio Political Declaration on Social Determinants of Health* [26] was mentioned in nine hits [30–38]. We also recognized that quantitative measures for assessing influence are limited given that texts may be prominent but not explicitly mentioned. To balance this challenge, we applied our knowledge of the field to better identify prominent and influential texts in these domains (e.g., major global report, political declaration). We elected to focus in at the institutional level, so we included different types of texts because WHO discourses are shared through different channels. Similarly, we did not restrict our selection by geographical region (e.g., regional- or country-levels). We also sought texts that explicitly cite and/or mention each other to uncover discourses across texts. This decision also aligns with CDA methodologically to assess interrelated systems of knowledge (please see Fig. 1 and discussed further below). Applying these criteria, we identified nine texts, and the final list of texts to be analyzed was discussed by the authorship team prior to analysis commencing.

These nine texts included in the CDA listed in Table 1, begin from the CSDH’s final report, *Closing the gap in a generation*, and include reports providing an overview on issues, a conference, a tool, and a user manual, and includes a political declaration. The selected texts are interrelated, which is most noticeable when assessing cross-references, as shown in Fig. 1. A linkage between texts that reference each other is depicted by a thick, solid line and instances in which a text is mentioned but not explicitly referenced are depicted by a dashed line. An example of this latter relationship is in *Our cities, our health, our future* (text 2 in Fig. 1), which does not cite the CSDH’s final report (text 1), but expresses that the CSDH’s framework guided the Knowledge Network on Urban Settings’ (KNUS) work [21]—this could be because the CSDH’s final report had not yet been published when the report was prepared. As another example, *Equity, social determinants and public health programmes* (text 3) cited the CSDH’s website, mentioned it in the forward, and had a full page dedicated to the CSDH’s report ([22] p. 292), but it did not explicitly cite the CSDH’s final report (text 1). Also, although the *Urban HEART User Manual* (text 5) did not explicitly cite the *Urban HEART: Urban Health Equity Assessment and Response Tool* key report (text 4), it is evidently based on this work. On the other hand, although *Hidden Cities: Unmasking and Overcoming Health Inequities in Urban Settings* (text 6) discusses Urban HEART and cites the *Urban HEART* report (text 4), it does not cite the user manual (text 5), resulting in no relationship in Fig. 1. Notably, neither the *Rio Political Declaration on Social Determinants of Health* (text 7) nor *Promoting health in the SDGs, Report on the 9th Global Conference for Health Promotion: All for health, health for all* (text 9) cited any sources, which results in no solid line relationships in Fig. 1. Overall, many of the texts mention each other, demonstrating interrelated systems of knowledge, which aligns with CDA methodology.

Selected texts are written by various authors, including the influential CSDH, and associated KNUS and Priority Public Health Conditions Knowledge Network (PPHCKN); the WHO Centre for Health Development (Kobe Centre), and in two instances co-authored with the United Nations Human Settlements Programme (UN-Habitat); and WHO more broadly. These texts describe health situations, but also include the *Urban HEART User Manual* and the *Rio Political Declaration on Social Determinants of Health*. Additional information on texts’ author(s) and select authorship acknowledgements is chronicled in Additional file 1.

## Results

The proceeding results demonstrate that (i) underpinning values are consistent in WHO texts’ approach to health equity, which aligns with the findings from Amri et al. on how scholars perceive the WHO’s approach to equity [11]; (ii) WHO texts reiterate that health inequities are socially constructed and mitigatable but leave the “causes of causes” vague, such as colonization, which may make policy efforts unfruitful without fully understanding what these are; (iii) despite expressing a distinction between health “inequities” and “inequalities,” there are several instances where WHO texts use “inequity” and “inequality” interchangeably across texts; (iv) WHO texts approach health equity broadly (e.g., including resources for health; determinants; outcomes or disparities in health and healthcare; consequences of specific diseases, conditions, or environments; allocation and utilization of resources; access to care or quality curative services; health opportunities and outcomes; and the organization of society); (v) depending on the specific WHO text at hand, health equity may be viewed as applicable throughout the life-course or intergenerationally, each of which has implications for policies and programs put forward; and (vi) WHO texts at times use vague or unclear language around how to tackle health inequities. These results are outlined in Table 2 and discussed in detail below.

**Table 2:** Overview of results

	Results
1	The underpinning values are consistent in WHO texts’ approach to equity
2	WHO texts reiterate that health inequities are socially constructed and mitigatable but leave the “causes of causes” vague, such as colonization, which may make policy efforts unfruitful without fully understanding what these are
3	Despite expressing a distinction between “inequities” and “inequalities,” there are several instances where WHO texts use “inequity” and “inequality” interchangeably across texts
4	WHO texts approach equity broadly (e.g., including resources for health; determinants; outcomes or disparities in health and healthcare; consequences of specific diseases, conditions, or environments; allocation and utilization of resources; access to care or quality curative services; health opportunities and outcomes; and the organization of society)
5	Depending on the specific WHO text at hand, health equity may be viewed as applicable throughout the life-course or intergenerationally, each of which has implications for policies and programs put forward
6	WHO texts at times use vague or unclear language around how to tackle health inequities

### Consistent underpinning values

The texts analyzed demonstrated alignment with Whitehead’s definition of health inequity [3], which was commissioned by the WHO. As stated above, Whitehead defines inequity in health as “differences which are *unnecessary* and *avoidable* … [and] considered *unfair* and *unjust*” [3]. For examples of excerpts that align with Whitehead’s language, please see Table 3. As a set, these links demonstrate the pervasive and ingrained nature of Whitehead’s definition and consistent underpinning values.

**Table 3:** Sample excerpts demonstrating alignment with Whitehead’s definition

Text	Sample expression(s) of health in/equity
1) *Closing the gap in a generation* [2]	“Where systematic differences in health are judged to be avoidable by reasonable action they are, quite simply, unfair. It is this that we label health inequity. Putting right these inequities – the huge and remediable differences in health between and within countries – is a matter of social justice. Reducing health inequities is, for the Commission on Social Determinants of Health (hereafter, the Commission), an ethical imperative” [2]. Further, the CSDH outlined much of the global burden of disease as “avoidable” and “unacceptable,” thus, “inequitable” [2]
2) *Our cities, our health, our future* [21]	Cited the WHO and identified equity as “the absence of unfair and avoidable or remediable difference in health among population groups defined socially, economically, demographically and geographically” [21]
3) *Equity, social determinants and public health programmes* [22]	Referenced the CSDH and stated that “where systematic differences in health are judged to be avoidable by reasonable action they are, quite simply, unfair. It is this that we label health inequity” [22] and indicated that “socioeconomic inequities include differences that are ‘systematic, socially produced (and therefore modifiable) and unfair’” [22]
4) *Urban HEART: Urban Health Equity Assessment and Response Tool* [23]	“… three features, when combined, turn a mere difference in health into an inequity in health. A difference in health that is systematic, socially produced (and, therefore, modifiable) and unfair is an inequity in health” [23]. Additionally, cited the CSDH’s definition of health inequity: “‘Where systematic differences in health are judged to be avoidable by reasonable action they are, quite simply, unfair. It is this that we label health inequity.’ The Commission adds: ‘Putting right these inequities – the huge and remediable differences in health between and within countries – is a matter of social justice’” [23]
5) *Urban HEART User Manual* [24]	“Equity is an ethical concept of social justice or fairness. It comprises two elements: horizontal equity, which is the equal treatment of equals; and vertical equity, which is the unequal but fair treatment of unequals” [24]. Further, indicated that “… health inequities are systematic and unjust” [24] and an health inequity is a “difference in health that is systematic, socially produced and unfair” [24]
6) *Hidden Cities: Unmasking and Overcoming Health Inequities in Urban Settings* [25]	“… health inequities, which are defined as health inequalities that are systematic, socially produced (and therefore modifiable) and unfair” [25]. Further, “health equity is, above all, an issue of social justice, and an indicator of the ability of cities to provide their residents with the prerequisites for health and well-being, and to help them achieve fulfilment of their aspirations and capabilities” [25]
7) *Rio Political Declaration on Social Determinants of Health* [26]	“We reaffirm that health inequities within and between countries are politically, socially and economically unacceptable, as well as unfair and largely avoidable, and that the promotion of health equity is essential to sustainable development and to a better quality of life and well-being for all, which in turn can contribute to peace and security” [26]
8) *Global report on urban health: equitable, healthier cities for sustainable development* [27]	“Inequalities that are systematic and remediable are considered to be inequities, and are a manifestation of social injustice” [27] and “when such differences, or inequalities, are not random but are systematic, and not due to biologically determined factors but due to modifiable social factors, they are unjust inequities” [27]. Additionally adding “urban inequity is obviously unjust” [27]
9) *Promoting health in the SDGs, Report on the 9th Global Conference for Health Promotion: All for health, health for all* [28]	“Policies for health and social justice benefit the whole of society” [28] and “unacceptable health inequities require political action across many different sectors and regions” [28]. Additionally, “the SDG agenda provides all countries and sectors, including health and other development sectors, with a clear roadmap for action and an ethical imperative to leave no one behind” [28]

In *Equity, social determinants and public health programmes*, it is noted that “health equity is a moral position as well as a logically-derived principle” and that “there are both political proponents and opponents of its underlying values” [22], reiterating this understanding of the values inherent in health equity. What comes across consistently is the notion that “health equity is social justice in health” [27] and that action on health inequities is rooted in the “principles of justice, participation, and intersectoral collaboration” [2]. In fact, in *Urban HEART: Urban Health Equity Assessment and Response Tool*, it was stated that “we know how to reduce inequities with known interventions and to not take action is unjust” [23]. Therefore, to advance and take action, actors must first ensure they have shared values, as some scholars believe shared values are required for political decision-making [39].

However, occasionally this overt social justice approach to health inequity does not come through in the texts. For instance, “an example of an inequitable abortion policy would be allowing individual medical practitioners to apply their own values to decisions about whether women should have access to safe abortion or making safe abortion services accessible to rich women but not poor women” [22]. Although the latter example around making abortion services only accessible to rich women aligns with Whitehead’s definition of health inequity, the former example around allowing individual medical practitioners to apply their own values to decisions does not necessarily constitute a systematic *difference* that is unjust or unfair, without additional contextual factors at play (e.g., if this is the only medical practitioner in the area). Or as another example of the social justice approach to health inequity being less clear is in a reference to the WHO’s Framework Convention on Tobacco Control (FCTC) as an “equity lever” for “conferring power on the many developing countries that otherwise would not be able to stand up to the tobacco industry” [22]. Though one could argue that although the FCTC is of assistance, it does not necessarily mitigate unjust and unfair systematic differences in health.

### Socially constructed and mitigatable

All assessed texts shared an understanding that health inequity is not a “condition of nature” or “randomly assigned”, whether explicit or implicit. Instead, health is “shaped by deeper social structures and processes … produced by *policies* that tolerate or actually enforce unfair distribution of and access to power, wealth, and other necessary social resources” (emphasis added; [2]), and similarly, “how health is distributed within a population is foremost a matter of *fairness in economic and social development policy*” (emphasis added; [22]). In addition to noting that health inequities result from *policy*, there is mention of the need for “broad and integrated interventions that address the *underlying causes of inequity* that result in poorer health and worse health outcomes” (emphasis added; [21]) or “focus[ing] on the ‘causes of the causes’” [2]. However, what constitutes these “underlying causes of inequity” is largely left vague; for example, “health inequities result from unequal distribution of power, prestige and resources among groups in society” [22], “the underlying causes of the causes of inequities are often associated with social status, discrimination or exclusion” [21], and “health inequities are the result of the circumstances in which people grow, live, work and age, and the health systems they can access, which in turn are shaped by broader political, social and economic forces” [25]. Although mitigating health inequities through policy is needed, this focus will not necessarily address the underlying causes of health inequities if the WHO is unsure of what they are or is unwilling to name and target them. Understandably, naming these causes can be complex, and the causes of health inequities can be wide-ranging; however, by not formally recognizing these causes, there is no subsequent impetus to address them. In other words, if causes are named, organizations and individuals should then act and disrupt the status quo, which, at present, benefits them. As such, there is little incentive for those in positions of power or privilege, such as the WHO, to name and target causes of causes, which may be a result of relying on influencers who preclude the naming of causes of causes. This lack of naming the causes of causes exists despite expressing that improving health “*depends* on understanding the causes of these inequities and addressing them” [22]. Similarly, what constitutes good policy [22], although well-intentioned, may be subjective (e.g., does it entail policy that is evidence-informed, addresses the SDH, promotes universal access to resources that may improve health, or does it entail something else altogether?).

But with so much attention paid to policy, arguably, sufficient attention should also be allocated to other areas, such as governance. Is it that adopting better governance is difficult to achieve or not well-defined? Is it that improvements in health indicators but not health equity are prioritized in WHO texts? Or are there other reasons?

Questions emerge as we assess the language employed when defining health in/equities. For example, when distinguishing between health inequities that are “systematic differences in health [that when] judged to be avoidable by reasonable action they are, quite simply, unfair” [2], the inclusion of “avoidable by reasonable action” draws debates around what constitutes both “avoidable” and “reasonable,” with the former discussed by Bambas and Casas [40]. And in terms of the latter, the CSDH identifies various actions across different levels, including community mobilization, multisectoral action, and progressive taxation, but whether these actions are reasonable is likely dependent on individual discretion and their context (e.g., multisectoral action may be deemed “reasonable” in one’s jurisdiction if there is political will and established systems for liaising that enable multisectoral action [41], as opposed to more innovative multisectoral approaches not previously undertaken [42]).

Similar questions emerge from statements such as “most individuals and societies, irrespective of their philosophical and ideological stance, have limits as to how much unfairness is acceptable” and “[health inequities] are avoidable, in that there are plausible interventions” [22]. The use of “unfair” is addressed in *Urban HEART Urban Health Equity Assessment and Response Tool*, which indicates that “although ideas about what is unfair may differ to a certain degree from place to place, there is much common ground. For example, it would be widely considered unfair if the chance of survival was much poorer for the children of some socioeconomic groups, compared with that of others” [23]. But given the global scope of the WHO’s work, there remains potential for different cultural and political understandings around what constitutes fairness.

### Differentiating health inequity from inequality

As established above, health inequities are thought to be unnecessary, avoidable, unfair, and unjust. These conditions help differentiate health inequities from health inequalities in the texts analyzed, which are thought to be measured differences that are not unfair and unjust [6]. Consider the following statement from *Hidden Cities: Unmasking and Overcoming Health Inequities in Urban Settings*: “some health inequalities are not health inequities. For example, death rates among people in their eighties are higher than those among people in their twenties, but this is not a socially produced, unfair health inequity. Rather, it is the result of the natural biological process of ageing” [25].

This distinction between health inequities and health inequalities is also expressed in *Hidden Cities: Unmasking and Overcoming Health Inequities in Urban Settings* [25], in which health inequalities are defined as “simply differences in health between groups of people. These differences might be due to non-modifiable factors such as age or sex, or modifiable factors such as socioeconomic status.” Similarly, health inequities are defined as being “systematic, socially produced (and therefore modifiable) and unfair” [25]. In large part, this differentiation did align with the use of these terms in the texts analyzed. For example, in discussing the equity gauge approach, the CSDH [2] discusses the gauge as an approach to address “unfair disparities in health and health care.”

Given the emphasis placed on health inequities being unjust and unfair, there are instances where “inequalities” is used where the more appropriate term would be “inequities.” Instances of the occasional interchanged use of these terms include indicating: that “health inequalities in urban areas need to be addressed in countries at all income levels” [21], that “promotion of exclusive breastfeeding can still contribute to reducing mortality inequalities, because fewer than half of the poorest children in low- and middle-income countries are exclusively breastfed” [21], that “relying on city averages, rather than examining differences between neighbourhoods and urban subgroups, has further obscured inequalities within cities” [25], that “large inequalities have emerged between city dwellers, and urban slums have become a feature of many cities” [25], and that “a common theme across all global initiatives on health in cities has been the need to tackle inequalities in health” [27]. Given these outlined examples, by writing “inequalities” instead of “inequities,” the focus is then on the measured difference rather than their unjust and unfair nature and, thus, addressing these health inequities. Although the selection of “inequalities” in place of “inequities” could be based on geographic differences in terminology use or editorial changes, this does not align with what is presented in each of the texts analyzed (see Table 3).

WHO texts have also used these terms interchangeably when applied to outcomes, which is illustrated by contrasting statements by Blas et al. [22]: “in the United Kingdom, alcoholic cirrhosis used to be a rich man’s disease [43], but there was a shift (in England and Wales) in the relative index of inequality in male liver cirrhosis mortality by social class from 0.88 in 1961 to 1.4 in 1981 (i.e., from lower to higher mortality in lower socioeconomic categories),” with an example provided to explain health inequities “[i]n Glasgow, Scotland, male life expectancy varies from 54 to 82 years, depending on the part of the city in which the person lives” [25]. Additional sample excerpts are noted in Table 4. Although these statements are not inaccurate, they do not align with WHO texts’ expressed position that these health inequalities are unjust and unfair and thus, health inequities.

**Table 4:** Additional sample excerpts demonstrating the interchanged use of health inequities and inequalities

Sample excerpts
• “inequalities in health in urban settings reflect, to a great extent, inequities in economic, social and living conditions” [21]
• “economic inequalities are often associated with health inequalities” [21]
• “health inequity leads to a gradient of inequalities in most societies at all levels of economic development” [21]
• “current and emerging eco-friendly approaches to town planning, housing design and workplace developments need to be systematically applied in order to minimize health inequalities in the future” [21]
• “it has been argued that the relatively stringent alcohol policies of the Nordic countries have contributed to holding down health inequalities there” [22]
• “a comparison of the two largest cities, Tokyo and Osaka, revealed that over half of the 23 wards that form the urban core of Tokyo have lower levels of mortality than the national average; in contrast, only one of the 24 wards in Osaka had lower levels of mortality than the national average. The range in ward-level mortality was also much wider in Osaka than in Tokyo. These examples illustrate place-based inequalities” [27]
• “there was a consistent pattern of inequality in total mortality in almost all cities, with mortality increasing in parallel with socioeconomic deprivation” [27]
• “inequalities in skilled birth attendance” [27]
• “relative inequalities in access to piped water are particularly high. Households in the richest quintile are 2.7 times more likely to have access to piped water compared to the poorest 20% households” [27]
• “it is difficult to isolate the contribution of the programme to observed declines in excess winter deaths and child accidents in the home, given the large number of other projects tackling poverty and health inequalities in the city” [27]
• “the UHI is a single, composite metric that can be used to measure and map the inequalities in health determinants and outcomes in urban areas” [27]
• “addressing food safety inequities involves evaluating the effectiveness of interventions in reducing inequalities in food safety” [22]

Interestingly, there is a footnote mention of the use of “inequities” over “inequalities” in health in the violence and unintentional injury chapter in *Equity, social determinants and public health programmes* [22]. After indicating that “injuries are a major contributor to inequities^2^ in health,” the footnote clarifies that “there are different views on the use of language. The authors of this chapter had originally inclined to the use of ‘inequalities’ in health, but, in the interests of consistency, have adopted the terms used elsewhere in this volume” [22].

Although the examples of interchanged use noted above and in Table 4 may be due to a lack of globally accepted differentiation [44] or various authors working on texts who may use the terms in different ways, a lack of understanding of the difference between these two terms or need to distinguish them, little attention paid to nuance by report writers, among many other potential reasons, it appears there is an intentional switching of terms at times. For example, despite the *Global report on urban health: equitable, healthier cities for sustainable development* indicating that data across 102 countries was analyzed to determine “health and health inequities,” one objective of this analysis of 102 countries was to “identify patterns, magnitudes and trends of health *inequalities* in urban settings at the national level on key health and social determinants of health indicators,” whereas another objective was to “identify health *inequities* in selected cities where sample sizes were sufficiently large and data were reliable” (emphases added; [27]). Similarly, the “widespread use of socioeconomic stratification variables, in particular asset quintiles, allows monitoring *inequities* in coverage and impact indicators on a regular basis. Most surveys are representative for subnational areas, thus also allowing the study of regional *inequalities*” (emphasis added; [22]). Further, “addressing food safety *inequities* involves evaluating the effectiveness of interventions in reducing *inequalities* in food safety” (emphasis added; [22]). The rationale for this usage of the two terms is unclear. We can speculate that in these instances “inequalities” is specifically referring to the measured difference that is unjust and unfair; however, this is not always in alignment with how the terms are used.

Additionally—although different from interchanging terminology—the use of adjectives when referring to health inequities can muddy waters. Consider the following sentence: “when such differences, or inequalities, are not random but are systematic, and not due to biologically determined factors but due to modifiable social factors, they are unjust inequities” [27]. This use of “unjust” prior to “inequities” raises questions about whether just inequities exist. Similarly, the use of “unacceptable” in “unacceptable health inequities require political action across many different sectors and regions” [28] raises questions around what constitutes an acceptable health inequity. And lastly, the inclusion of “socially determined” prior to “health inequalities” in “the attention to socially determined health inequalities is a common feature of the observatories, which necessitates an intersectoral and community inclusive approach in both generating and applying the data” [27] raises questions on how “socially determined health inequalities” differ from health inequities. Evidently, this language could be more precise, for example, by using “politically determined” as opposed to “socially determined.”

### Health inequities in what?

Drawing on the question of “health inequities in what?”—adapted from the question posted by Sen: “equality of what?” [19]—may lead to better understanding what discussions of health equity can center around and ultimately, potentially allow for more deliberate action [45]. Blas et al. [22] indicate that “three principal measures are commonly used to describe health inequities,” which are health disadvantages, health gaps, and health gradients. However, health equity is presented in the analyzed texts in terms of the unequal distribution of various “things,” including resources for health; determinants; outcomes or disparities in health and healthcare; consequences of specific diseases, conditions, or environments; allocation and utilization of resources; access to care or quality curative services; health opportunities and outcomes; and the organization of society. Select excerpts are presented in Table 5 to illustrate these various aspects. Carefully specifying and considering the question “health inequities in what?” is crucial because it can entail differing philosophical perspectives. Rawlsian theory would seek to distribute resources based on individual need, whereas Sen’s theory recognizes that the provision of goods will not result in the same outcome for individuals, and thus focuses on maximizing individual capabilities to function or equalizing capabilities among individuals [43, 46]. Each of these theories provide different interpretations for how to strive for health equity, which must be taken into consideration. Ultimately, this demonstrates the need to clarify what discussions of health equity may be referring to more precisely.

**Table 5:** Sample excerpts of how health equity has been discussed

Sample excerpts
• “disparities in health and health care” [2]
• “resources for health” [2]
• “key aspects of nutrition and health equity: availability, accessibility, and acceptability” [2]
• “equity issues in urban health and health impacts” [21]
• “inequities in the determinants, outcomes and consequences of [cardiovascular disease and
diabetes]” [22]
• “allocation of resources to prevention and control of [cardiovascular disease]” [22]
• “inequities in tobacco use” 35]
• “equity in access and financial protection for the poor” [22]
• “unfair differences, or inequities, in health opportunities and outcomes” [24]
• “health equity implies that everyone has a fair opportunity to attain their full health potential” [25]
• “health determinants and health outcomes” [25, 27]
• “good health and affordable access to the health care” [27]
• “health-care utilization” [27]
• “health risk exposure, health behaviours, access to health care and health outcomes” (36)
• “inequality or disadvantage they were born into, and by promoting equality of opportunity in employment and education” [27]
• “in access to quality curative services” [22]
• “fair access to public services and work towards universal health coverage” [28]
• “social determinants (ethnicity, gender, education, migration, trade, urbanization, demographic factors and poverty)” [22]
• “inequities in how society is organized” [2]
• “equity gap in the incidence or morbidity, mortality, candidacy for risk and access to effective treatment, or better, prevention” [22]

### Health equity as applicable throughout the life-course or intergenerational?

Select texts used language around the intergenerational aspect of health inequity, whether stated directly (e.g., “this reinforces the inequities in the distribution of other health conditions and can carry important intergenerational consequences” ([22], p. 121)) or indirectly (e.g., “inaction has detrimental effects that can last more than a lifetime” ([2], p. 59) and “this affects children as well, as whole families are bonded under the kamaiya system” ([2], p. 77)). However, this intergenerational way of thinking about health equity does not align with select expressions of public health action, in particular, targeting health through the *life-course*, which has been encouraged in WHO texts (e.g., “at the heart of it all is the challenge of health equity–ensuring that all people have the opportunity to achieve good health and affordable access to the health care they need throughout the life-course” ([27], p. 30)). These differing discourses are notable, as the policy and program actions that WHO texts are presenting as potential solutions do not explicitly discuss intergenerational aspects. By considering health equity as intergenerational, actions need to align with this discourse, including efforts that target sustained benefits over generations and associated long-term evaluations of efforts.

### Vague or unclear approaches to improving health equity

Despite the texts’ focus on health equity and their aim to bring about change, much is frequently left unstated about how to proceed. This is best illustrated through commonplace language around the need for an “equity lens,” which was simply stated and without further explanation. One example that drew on the language of “equity lens” that did in fact provide specificity was indicating the need to

build capacity in applying the equity lens to the monitoring instruments and methodologies themselves. For example, population sampling frames are often based on physical address area codes, excluding the many vulnerable, informal settlers who do not have an official physical address. The public health community needs to be highly critical of its monitoring and surveillance tools and methodologies, to apply the equity perspective to how we measure impacts and gather data, and to strive to design monitoring mechanisms that are inclusive and equitable [22].

Although helpful in expressing what is meant by an “equity lens,” does it entail that by simply ensuring vulnerable populations are counted, health equity can be achieved? It would be safe to assume that this is not so, but questions such as this one remain around who would be targeted, to what extent, targeted for what, etc. Arguably, this text’s broader focus on identifying priority public health conditions may skew discussions of an “equity lens” to be more pragmatic and focused on methodologies.

On the contrary, the WHO’s Urban HEART was identified as being able to provide an “equity lens” [23] and more specifically in one instance, in “policy-making and resource allocation decisions” [24]. Through Urban HEART’s actionable identification and mapping of health inequities, it perhaps fulfills this aim. However, Urban HEART’s identification of implications for governments adopting a “health equity lens” entailed “governments will adopt a health equity lens when planning resource allocation” [24]. This application of a “health equity lens” similarly leaves questions unanswered (e.g., is this lens always in reference to resource allocation? What steps should be taken in allocating resources to maximize health equity?).

Other instances of unclear language include indicating that “health equity can be considered as a reliable way to measure and monitor how well a city is meeting the needs of its residents” [25] and “epidemiology and research to add … an equity dimension” [22], but how this may be determined in practice remains vague. An example of this is provided in the report by the CSDH: “Health equity impact assessment is one of the tools recommended by the Commission … to help decision-makers to systematically assess the potential impact of policies, programmes, projects, or proposals on health equity in a given population with the aim of maximizing the positive health equity benefits and minimizing the potential adverse effects on health equity” [2]. For instance, how can positive health equity benefits be maximized when understandings of ideal health equity outcomes may vary across stakeholders (e.g., improve those within the bottom fifth wealth quintile vs. improve the health of all in a given community [47])?

Thus, vague language like “implementing pro-equity policy and planning” [25], “tak[ing] into account health equity” [2], and “integrat[ing] equity, as a priority within health systems, as well as in the design and delivery of health services and public health programmes” [26] is limited in its ability to guide practice without further details on what striving for health equity means or looks like.

## Discussion

To improve public policymaking, clear operational definitions are required to ensure that objectives, targets, and priorities can be established and, accordingly, to assess progress [44]. Most obviously, this relates to the interchanged use of health “inequities” and “inequalities” in WHO texts, which was finding (iii) noted alongside other findings in Table 2. Without the acceptance of globally appropriate definitions, operationalizing action on health inequities can be unclear and objectives will vary depending on the parties involved. Moving beyond setting objectives, the varying discourses around health equity can also shift action accordingly. By critically assessing discourses, we can challenge current views, norms, and operations that are held by individuals in policy and practice. In taking the unwillingness to name causes of causes as an example, work is shifted away from addressing fundamental issues. Similarly, in targeting health inequity throughout the life-course, rather than intergenerationally, sought actions will inevitably differ. This is particularly noteworthy when considering how the policy objectives of large, multilateral global health organizations, such as the WHO, differ from member state priorities in recent years, where the latter tend to be shorter-term priorities [48]. As another example, instead of discussing applying an “equity lens,” what this entails can be described at the outset. This novel study may also guide scholarship to examine other organizations’ work more critically. As noted by Sen [49], considering the concept of health equity can lead to questions and perspectives that work to enrich the abstractness of equity in general. Thus, this work paves the way for further inquiry into the operationalization of complex key terms or those with embedded values.

### Limitations

Although this study was not designed to define health equity and determine how to best act on it, it does seek to uncover the multiple discourses embedded in WHO texts to shed light on how these may result in differing actions. However, one potential limitation arises from the ability of the selected texts to represent the WHO as a singular, monolithic, or unitary organization. For instance, texts produced by headquarters may not necessarily reflect the views or positions of all WHO regions—with note that these analyzed texts are all in English—or result in regions accordingly following through with associated practices. Regions may take their own direction on work, reflecting the differing sociopolitical contexts in which they operate, with one example being the WHO Regional Office for Europe undertaking the “Inequalities in health system performance and social determinants in Europe—tools for assessment and information sharing” project with the European Commission [50]. Although out of scope for this study, investigations are warranted into assessing how conceptualizations of health equity differ across specific regions; programmatic areas, such as Healthy Cities, in addition to less health equity-imbued areas, such as the “big three” [51] and perhaps “big four” with COVID-19; and the World Health Assembly’s workings as the decision-making body of the WHO. Similarly, because individuals are responsible for writing texts, it may be difficult to ascertain if ideas are associated solely with the author(s) or reflect the views of the broader organization or organizations responsible for the report(s) (e.g., attributable to the WHO and/or UN-Habitat for two joint reports in the study). Although all texts included in this study are WHO texts, further information on each texts’ respective author information is specified in Additional file 1. However, with respect to this potential limitation, it is noteworthy to consider that individuals’ views reflected in these texts devise broader WHO policy and practice [14]. And similarly, institutions follow select actions by way of their individual actors [14]. Therefore, although this may be considered a limitation by some, it is important to recognize that individuals inherently constitute organizations and the ways in which they operate. In addition, these texts are largely interrelated, as demonstrated in Fig. 1. Irrespectively, whether the text can be attributed to individual authors or the WHO more broadly, it is noteworthy that these texts shape broader global policy and practice.

## Conclusions

As far as we are aware, our study is the first to assess empirically how the WHO approaches health equity. Our findings are an important first step toward addressing this critical gap in knowledge [52] for policymaking and scholarship. Our aim is to go beyond earlier efforts, including the CSDH’s final report, which some critiqued for its limited policy guidance and over-emphasis on problems as opposed to solutions [53]. These findings therefore can be applied by the WHO in planning, implementing, and evaluating initiatives, particularly when considering the wide applicability across numerous vertical programs in global health, which are also prioritized by member states [48]. These findings also have implications for policy and program work more broadly outside of the WHO.

Despite the WHO and UN-Habitat identifying a “prerequisite to action” as “developing a common vision for health and health equity” [25], at present, this appears to be missing within select WHO texts, as demonstrated in this study, and among WHO actors involved in Urban HEART [54]. This may be partially attributable to health equity being a “relatively new concern and … not universally applied in public health practice as an operational concept” [21]. However, with ongoing WHO commitments to “improving the health and well-being of all” (e.g., Fifth Health Sector Directors’ Policy and Planning Meeting for the WHO African Region [55]) and desire to “integrate equity, as a priority within health systems, as well as in the design and delivery of health services and public health programmes” [26], what that means and what it could look like needs to be further interrogated. This is particularly important within the context of the COVID-19 pandemic. COVID-19 has afforded lessons in policy development, including moving away from short-sighted solutions to ensuring policy is strategic and focused on equity [56]. Thus, this opportunity to pinpoint what health in/equity means and how it can be acted on in the long term can be seized, given that COVID-19 may provide an opportunity to refocus on the SDH and health equity [57]. As such, the results of this study should be utilized to consider what health equity entails in global and public health and policy work and drawn on in determining appropriate courses of action. 

## Supplementary Information

**Additional file 1.** Texts’ author(s) and select authorship acknowledgements. This file contains a table that details the title of each text analyzed, information on the text’s author(s), and select authorship acknowledgements as noted in each text.

## Data Availability

All data analyzed during this study are listed in this published article in Table 1.

## Abbreviations

CDA: Critical discourse analysis
CSDH: Commission on the Social Determinants of Health
SDH: Social determinants of health
Urban HEART: Urban Health Equity Assessment and Response Tool
WHO: World Health Organization
