# Supplementary material for: Health promotion, the social determinants of health, and urban health: what does a critical discourse analysis of World Health Organization texts reveal about health equity?
Source: BMC Glob Public Health. 2023 Dec 1;1:25. doi: 10.1186/s44263-023-00023-4 (PMC11622871; doi:10.1186/s44263-023-00023-4)
Supplement: Supplementary file 1 — Additional file 1. Texts’ author(s) and select authorship acknowledgements. This file contains a table that details the title of each text analyzed, information on the text’s author(s), and select authorship acknowledgements as noted in each text. [file 44263_2023_23_MOESM1_ESM.docx]

**Additional file 1.** Texts’ author(s) and select authorship acknowledgements.

| **Text** | **Author(s)** | **Select authorship acknowledgements** |
| --- | --- | --- |
| **1)** *Closing the gap in a generation* | WHO Commission on Social Determinants of Health (CSDH) | - “This publication contains the collective views of the Commission on Social Determinants of Health and does not necessarily represent the decisions or the stated policy of the World Health Organization”. - “The work of the Commission was championed, informed, and guided by the Chair of the Commission and the Commissioners. Report writing team: Michael Marmot, Sharon Friel, Ruth Bell, Tanja AJ Houweling, and Sebastian Taylor. The team is indebted to all those who contributed to the development of the report, including Commissioners, Knowledge Networks, country partners, civil society facilitators, and colleagues in the World Health Organization (WHO), Geneva”. - Please see Acknowledgements page for further details. |
| **2)** *Our cities, our health, our future* | WHO’s CSDH’s Knowledge Network on Urban Settings (KNUS) | - “The views expressed in this publication are those of the authors and do not necessarily represent the decisions, policy or views of WHO or Commissioners”. |
| **3)** *Equity, social determinants and public health programmes* | WHO’s CSDH’s Priority Public Health Conditions Knowledge Network (PPHCKN)  Edited by Erik Blas and Anand Sivasankara Kurup | - “The authors of various chapters of the book are: Jens Aagaard-Hansen, Awa Aidara-Kane, Amitava Banerjee, Fernando C. Barros, Erik Blas, Claire-Lise Chaignat, Joanne Corrigall, Annette David, Chris Dye, Katharine Esson, Christopher Fitzpatrick, Alan J. Flisher, Michelle Funk, Davidson Gwatkin, Sean Hatherill, Norman Hearst, Ernesto Jaramillo, Jean-Louis Jouve, Stella Kwan, Knut Lönnroth, Crick Lund, Pia Mäkelä, Shawn Malarcher, David Meddings, Shanthi Mendis, Les Olson, Vikram Patel, Anne-Marie Perucic, Poul Erik Petersen, Sophie Plagerson, Mario Raviglione, Jürgen Rehm, Helen Roberts, Gojka Roglic, Robin Room, Robert W. Scherpbier, Laura A. Schmidt, Anand Sivasankara Kurup, Nigel Unwin, Cesar G. Victora, David Whiting and Brian Williams”. - “Valuable inputs in terms of contributions, peer reviews and suggestions on various chapters were received from a number of WHO staff at headquarters, regional offices and country offices […]”. - “This book was commissioned by the Department of Ethics, Equity, Trade and Human Rights as part of the work undertaken by the Priority Public Health Conditions Knowledge Network of the Commission on Social Determinants of Health in collaboration with 16 of the major public health programmes of WHO […]”. - “The named authors alone are responsible for the views expressed in this publication”. - “This book was produced under the overall direction of Tim Evans (Assistant Director-General), Jeanette Vega, Nick Drager (former Directors of the Department of Ethics, Equity, Trade and Human Rights) and Rüdiger Krech (present Director of the Department of Ethics, Equity, Trade and Human Rights)”. |
| **4)** *Urban HEART Urban Health Equity Assessment and Response Tool* | World Health Organization, The WHO Centre for Health Development | - “The Urban Health Equity Assessment and Response Tool (Urban HEART) is a collective effort and has been jointly developed by the WHO Centre for Health Development, Kobe (Japan), in collaboration with regional offices of WHO, and city and national officials from across the world. Inputs from the teams in cities who pilot-tested the tool have been critical in the development of Urban HEART: Guarulhos (Brazil); Jakarta, Denpasar (Indonesia); Tehran (Islamic Republic of Iran); Nakuru (Kenya); State of Sarawak (Malaysia); Mexico City (Mexico); Ulaanbaatar (Mongolia); Davao, Naga, Olongapo, Paranaque, Tacloban, Taguig, Zamboanga (Philippines); Colombo (Sri Lanka); Ho Chi Minh City (Viet Nam). In particular, the enthusiasm of community groups in various pilot sites and their leadership in building broad-based support for applying the tool to address health inequities in their cities were vital. Their inputs and emphasis on a participatory approach have been a key building block of Urban HEART”. The development of the tool has also benefited much from the expertise of officials at WHO headquarters in Geneva, especially from the Noncommunicable Diseases and Mental Health Cluster and the Information, Evidence and Research Cluster. Finally, the 12 members of the Urban HEART Ad Hoc Advisory Group brought in a variety of expertise to contribute to the development of the tool. The advisors, a mix of academics, policy-makers and experts from international organizations, provided much-needed technical inputs and advice to strengthen both the scientific validity and the practical applicability of the tool”. |
| **5)** *Urban HEART User Manual* | World Health Organization, The WHO Centre for Health Development | - “*The Urban Health Equity Assessment and Response Tool (Urban HEART) User Manual* is a collective effort and has been jointly developed by the World Health Organization (WHO) Centre for Health Development, Kobe (Japan), in collaboration with regional offices of WHO, and city and national officials from across the world. Inputs from the teams in cities who pilot-tested the tool have been critical in the development of the Urban HEART User Manual: Guarulhos (Brazil); Jakarta, Denpasar (Indonesia); Tehran (Islamic Republic of Iran); Nakuru (Kenya); State of Sarawak (Malaysia); Mexico City (Mexico); Ulaanbaatar (Mongolia); Davao, Naga, Olongapo, Parañaque, Tacloban, Taguig, Zamboanga (Philippines); Colombo (Sri Lanka); Ho Chi Minh City (Viet Nam). In particular, we would like to acknowledge the role of community groups in various pilot sites for their enthusiasm and leadership in building broad-based support for applying the tool to address health inequities in their cities. Their inputs and emphasis on a participatory approach is a key building block of Urban HEART. Acknowledgement is also due to the Centre for Research on Inner City Health (Toronto, Canada) for their contribution in writing the User Manual based on inputs from various stakeholders”. |
| **6)** *Hidden Cities: Unmasking and Overcoming Health Inequities in Urban Settings* | World Health Organization, The WHO Centre for Health Development, and United Nations Human Settlements Programme (UN-Habitat) | - “The joint UN-HABITAT/WHO report *Hidden cities: unmasking and overcoming health inequities in urban settings* is the result of an intensive collaboration between the UN-HABITAT head office in Nairobi, Kenya, and the World Health Organization”. - Please see Acknowledgements page for list of individual contributors. |
| **7)** *Rio Political Declaration on Social Determinants of Health* | World Health Organization | - “Invited by the World Health Organization, we, Heads of Government, Ministers and government representatives came together on the 21st day of October 2011 in Rio de Janeiro to express our determination to achieve social and health equity through action on social determinants of health and well-being by a comprehensive intersectoral approach”. |
| **8)** *Global report on urban health: equitable, healthier cities for sustainable development* | World Health Organization, The WHO Centre for Health Development, and United Nations Human Settlements Programme (UN-HABITAT) | - “The report could not have been possible without the contributions from many WHO Departments, units, and Regional Offices, from UN-Habitat, and from over 150 experts outside of WHO” (30). - “This report was developed under the leadership of Marie- Paule Kieny, WHO Assistant Director-General of Health Systems and Innovation and Alex Ross, Director of the WHO Centre for Health Development, in partnership with UN-Habitat with appreciation to Oyebanji Oyelaran- Oyeyinka, Director of Regional Office for Africa, UN- Habitat. Paul Rosenberg and Amit Prasad coordinated the development of this report. The principal writers of this report were Paul Rosenberg, Megumi Kano, Isobel Ludford, and Amit Prasad. Thomson Prentice provided support for technical writing and AvisAnne Julien was the technical editor for the report. Additional data analysis for the report was supported by Dajun Dai, Cecilia Vidal Fuertes, Richard Rothenberg, Christine Stauber, Scott Weaver, and Doohee You. Accurat S.R.L. developed the layout, design and data visualisations for the report. Mariko Yokoo provided administrative support. This report benefited greatly from the inputs of many WHO and UN-Habitat colleagues”. - “All chapters are based on contributions from WHO and UN-Habitat departments, programmes and partnerships as well as other United Nations agencies and academic institutions […]”. - Please see Acknowledgements (30) for information on the Steering Committee, Scientific Advisory Committee, Contributors, and Research Assistants. |
| **9)** *Promoting health in the SDGs, Report on the 9th Global Conference for Health Promotion: All for health, health for all* | World Health Organization | - “WHO acknowledges the leadership and support of the Conference Organizing Committee and the Scientic Committee, as well as the contributions of Professor Ilona Kickbusch who prepared the first draft of this report. We also acknowledge the contribution of the health promotion community, e.g. public health professionals, academics, researchers, government leaders, policy-makers and advocates, for their technical contributions during the various activities and discussions of the conference. The conference was supported by WHO staff at the headquarters, regional and country levels”. - Of note, signing of the Shanghai Mayors’ Consensus on Health Cities took place on Day 1. |
